# What Should a Clinician Do When Spreading Depolarizations are Observed in a Patient?

**DOI:** 10.1007/s12028-019-00777-6

**Published:** 2019-07-23

**Authors:** Raimund Helbok, Jed A. Hartings, Alois Schiefecker, Baptiste Balança, Sharon Jewel, Brandon Foreman, Ari Ercole, Ramani Balu, Cenk Ayata, Laura Ngwenya, Eric Rosenthal, Martyn G. Boutelle, Eszter Farkas, Jens P. Dreier, Martin Fabricius, C. William Shuttleworth, Andrew Carlson

**Affiliations:** 1 Department of Neurology, Neurocritical Care, Medical University of Innsbruck, Anichstrasse 35, 6020 Innsbruck, Austria; 2 Department of Neurosurgery, University of Cincinnati (UC) College of Medicine, Cincinnati, OH USA; 3 Collaborative for Research on Acute Neurologic Injury, University of Cincinnati (UC) College of Medicine, Cincinnati, OH USA; 4 UC Gardner Neuroscience Institute, Cincinnati, OH USA; 5 Center for Stroke Research Berlin, Charité – Universitätsmedizin Berlin, Corporate Member of Freie Universität Berlin, Humboldt-Universität zu Berlin, and Berlin Institute of Health, Berlin, Germany; 6 Department of Anesthesiology and Intensive Care Medicine, Hospices Civils de Lyon, Hôpital Pierre Wertheimer, Lyon, France; 7 Lyon Neuroscience Research Centre, Lyon, France; 8 Centre Lyonnais d’Enseignement par la Simulation en Santé, SAMSEI, Université Claude Bernard Lyon 1, Lyon, France; 9 Department of Basic and Clinical Neuroscience, King’s College, London, UK; 10 Department of Neurology and Rehabilitation Medicine, University of Cincinnati (UC) College of Medicine, Cincinnati, OH USA; 11 Division of Anaesthesia, Department of Medicine, University of Cambridge, Cambridge, CB2 0QQ UK; 12 Department of Neurology, Perelman School of Medicine, University of Pennsylvania, Philadelphia, PA 19104 USA; 13 Neurovascular Research Laboratory, Department of Radiology, Harvard Medical School, Massachusetts General Hospital, Charlestown, USA; 14 Department of Neurology, Massachusetts General Hospital, Boston, MA 02114 USA; 15 Department of Bioengineering, Imperial College London, London, SW7 2AZ UK; 16 Department of Medical Informatics, University of Szeged, Szeged, H-6720 Hungary; 17 Department of Neurology, Charité – Universitätsmedizin Berlin, corporate Member of Freie Universität Berlin, Humboldt-Universität zu Berlin, and Berlin Institute of Health, Berlin, Germany; 18 Department of Experimental Neurology, Charité – Universitätsmedizin Berlin, corporate Member of Freie Universität Berlin, Humboldt-Universität zu Berlin, and Berlin Institute of Health, Berlin, Germany; 19 Bernstein Center for Computational Neuroscience Berlin, Berlin, Germany; 20 Einstein Center for Neurosciences Berlin, Berlin, Germany; 21 Department of Clinical Neurophysiology, Rigshospitalet, Copenhagen, Denmark; 22 Department of Neurosciences, University of New Mexico School of Medicine, Albuquerque, NM 87131 USA; 23 Department of Neurosurgery, University of New Mexico School of Medicine, Albuquerque, NM 87131 USA

**Keywords:** Cortical spreading depression, Electroencephalography, Subarachnoid hemorrhage, Traumatic brain injury, Ischemic Stroke, Intracerebral hemorrhage, Outcome

## Abstract

The International Conference on Spreading Depolarizations (iCSD) held in Boca Raton, Florida, in the September of 2018 devoted a section to address the question, “What should a clinician do when spreading depolarizations are observed in a patient?” Discussants represented a wide range of expertise, including neurologists, neurointensivists, neuroradiologists, neurosurgeons, and pre-clinical neuroscientists, to provide both clinical and basic pathophysiology perspectives. A draft summary of viewpoints offered was then written by a multidisciplinary writing group of iCSD members, based on a transcript of the session. Feedback of all discussants was formally collated, reviewed, and incorporated into the final document which was subsequently approved by all authors.

## Introduction

The Co-Operative Studies on Brain Injury Depolarizations was established in 2003 as a clinical translational effort to determine whether spreading depolarizations (SD), as observed in animal models of neurological injury, could be recorded and related to outcomes after human brain injury. Using electrocorticographic (ECoG) monitoring from subdural electrode strips, those initial studies yielded the surprising result that SD incidences range from 55 to 90% in patients who have undergone neurosurgical treatment of brain trauma, ruptured aneurysms, intracerebral hemorrhage, or malignant hemispheric stroke. Those studies further revealed an association of SDs with markers of secondary brain injury, such as delayed cerebral ischemia, infarction expansion, brain edema, and excitotoxicity, as well as poor functional outcome. Based on this work, the predominant consensus is that SDs have an adverse impact, in at least some subset of patients. The accompanying discussion article highlights various considerations that may determine whether individual SDs are harmful or benign.

Given this background, clinicians who monitor patients for SDs are now confronted with the question as to what should be done, from both ethical and scientific perspectives, when faced with a critically ill patient who has neurologic deficits and ongoing SDs detected with ECoG monitoring. The question becomes even more pressing with (1) the emerging use of SD monitoring for clinical care, rather than research only, and (2) the increasing use in pre-hospital and neurointensive care of the analgesic and sedative, ketamine, a clinically available *N*-methyl-d-aspartate (NMDA) receptor antagonist. Several physiologic factors have been shown to influence SD, and ketamine is perhaps the leading treatment option to suppress them, based on published results from multiple centers. Yet, no studies to date have investigated whether interventions targeted to SD suppression have clinical benefit. Motivated by these considerations, a second discussion session was held at the International Conference on Spreading Depolarizations (iCSD; Boca Raton, Florida, September 22–24, 2018), as introduced in the accompanying article, to address the question, “What should a clinician do when spreading depolarizations are observed?” Discussants represented a wide range of expertise, including neurologists, neurointensivists, neuroradiologists, neurosurgeons, and pre-clinical neuroscientists, to provide both clinical and basic pathophysiology perspectives. This report is a summary of the viewpoints offered, based on a transcript of the session.

## SDs in the Clinical Context

Discussants from several centers attested to the clinical dilemma of whether and how to treat patients showing a severe course of SDs. Current approaches and responses to this problem vary across centers and even within centers depending on several factors. Some investigators reported that ketamine is frequently used as a response when clusters of repetitive SDs are detected on ECoG monitoring. Several other sites reported that the trigger to initiate treatment depends on the patient’s neurological exam or other multimodality monitoring parameters, such as changes in brain tissue oxygen tension, brain metabolism, or cerebral blood flow. Particularly challenging are cases where a patient may show improving performance on one aspect of a neurological exam (e.g., ability to follow commands), but demonstrate persistent or increasing numbers of SD events. Specific examples of this scenario were raised by a neurointensivist and a neurosurgeon at two different medical centers. Currently, invasive monitoring is often withdrawn in these patients despite ongoing SDs, and SD incidence or severity is not further considered. This common experience led to reflection on the purpose of invasive monitoring in neurointensive care and whether it is genuinely being used to inform treatment of disrupted brain physiology. It was pointed out that even when a patient is improving in terms of consciousness and motor responsiveness, SDs may still signal significant injury and cell death that are ongoing in brain regions that are not eloquent. The patient may recover, even to a good outcome score, yet have more subtle cognitive deficits that were perhaps preventable if neurointensive management were targeted to brain pathology.

## Treatment Options

It was noted that the use of ketamine as a primary candidate for treatment is a contentious topic in part because NMDA receptor antagonists have failed as neuroprotective agents in multiple clinical trials. However, it is possible that the failures were due to broad patient inclusion criteria, whereas SD monitoring on the other hand would allow mechanistic targeting and more selective patient inclusion. A further concern was that the ketamine dosing required for SD suppression is fairly high and that there may be neurotoxic and sedation-related adverse effects on other brain regions, which may go undetected and counteract any benefit of SD suppression. This idea was challenged, however, by one of the participants with experience in refractory status epilepticus, where ketamine doses well above 1.5 mg/kg/h (even up to 7.5 mg/kg/h) are used routinely and successfully. Though potential harm of these high doses is not fully known, this is a well-accepted practice in the field. Others contested that significant SD suppression can be obtained at much lower doses. Another concern is related to the discussion of whether SDs are universally harmful, and whether they might have beneficial effects in remote, uninjured regions (see accompanying discussion paper). The theoretical possibility was raised that ketamine could suppress SD in these more remote sites where they may be beneficial, but fail to suppress them in vulnerable areas of evolving injury. Indeed, several pre-clinical studies have shown that NMDA antagonists have reduced efficacy to block SDs in ischemic compared to normal tissue. Finally, there was concern that ketamine may blunt the hyperemia induced by SD in normal brain, which could confer benefit to the tissue. The counterpoint was raised that if the SD is inhibited by ketamine in the first place, then perhaps the hyperemia-blunting effect is a moot point, given the widely held theory that the hyperemic response is the compensatory mechanism for the enormous metabolic load of SD.

Other therapeutic options besides ketamine that were discussed revolved around optimization of systemic physiology. It was noted that both pre-clinical and clinical data have supported an association of SD with factors such as hypotension, decreased cerebral perfusion pressure, hypoglycemia, and elevated body and brain temperature. Several clinicians mentioned that, in the case of SD detection, a reasonable first-line response would be aggressive normalization of these variables. While achieving normal systemic physiology is a cornerstone of routine neurocritical care, it was noted that the “normal” target ranges for these variables can actually be quite wide, and that optimal values may be narrower and even differ between patients. One idea was that detection of SDs, as a potential indicator of brain injury progression, would lead to more aggressive or targeted management of these variables. Examples include fever control and elevation of cerebral perfusion pressure, even if already above the accepted minimum threshold. A concern was raised about “supranormal” responses, particularly related to glucose augmentation to levels higher than 180 m/gL. Similarly, detrimental effects of hypothermia, induced hypertension, and prolonged hyperoxia are possible. To date, no studies have investigated whether physiologic therapies can alter the course of SDs in patients.

While there are potential risks and benefits with any treatment, there was general agreement, given the preponderance of current data supporting a harmful role of SDs in some patients, that clinical studies of interventions are warranted and needed. Thus, a major theme of the discussion was how to design an interventional clinical trial, as summarized in the following sections.

## Patient Population

There was acknowledgement that patients who undergo ECoG monitoring are extremely diverse, and different approaches, both in terms of clinical care and research priorities, may vary along this spectrum. Malignant hemispheric stroke patients appear to almost universally experience SD and have generally poor outcomes due to the severity of the baseline condition. With “little to lose and much to gain,” it was suggested that this group might be appropriate for a safety or feasibility study. Aneurysmal subarachnoid hemorrhage was discussed as an attractive disease for a neuroprotective interventional study, considering the well-recognized secondary phase of delayed cerebral ischemia (DCI). Since SD is involved in DCI pathogenesis, DCI could serve as a relevant study endpoint. Furthermore, its delayed nature allows documentation and study of its development, as well as time for early intervention. Most likely, a feasibility trial should be conducted first before a large multicenter outcome study. Traumatic brain injury was also discussed as a priority for research, considering the disease incidence and patient availability. A drawback of brain trauma, however, is its wide heterogeneity in causes, patient demographics, and intracranial pathologies. There was agreement that any study should aim to focus on a specific, more homogeneous population. One suggestion, based on the work presented at this iCSD meeting, was patients with chronic subdural hematoma. Ongoing brain trauma registries such as TRACK-TBI and CENTER-TBI were discussed as important efforts toward improved definition of patient subgroups for therapeutic targeting. Intracerebral hemorrhage was proposed as a more homogenous disease; however, indications for surgery remain unclear for this condition.

## Precision Medicine Versus Generalizability

Another key topic discussed in trial design was the role of “precision medicine” approaches, as opposed to broadly generalizable studies. There was a strong opinion from some that the use of SD monitoring would be highly desirable in any interventional study, both to identify appropriate patients and to assess response to treatment. Proponents of this “precision medicine” approach argued that selecting patients with SDs, or a high SD burden, would identify the group most likely to benefit from therapy, and would further match the control group to the corresponding outcome risk. It was noted that previous failed trials of NMDA receptor antagonists did not have such selection. On the other hand, it was considered that only a small percentage of patients likely to experience SDs have a clinical need for surgery, which allows the placement of ECoG monitors. Thus, use of SD monitoring would be very restrictive and would present a challenge for recruitment of adequate numbers that may be needed in an interventional study. Inclusion of all patients with severe injury, or even those with more moderate injuries, who may also be at risk for SD, would be more feasible. Nonetheless, the true incidence and burden of SDs in these other populations are unknown, and it was cautioned that SD incidences and burden should not be extrapolated from surgical cases to all severity grades of the respective diseases. Extrapolation of treatments, therefore, is similarly troublesome.

One approach discussed to address the problem of generalizability was the development of noninvasive methods for SD diagnosis. This has emerged as an important topic at the iCSD meeting, with most efforts focused on scalp electroencephalogram (EEG) and, to a lesser extent, near-infrared spectroscopy. Short of a direct diagnostic, it was also suggested that surrogate markers or predictors of SD would be useful, particularly in the context of patient selection for clinical trials. Such markers could be population based, such as demographics, injury characteristics, or severity grading, to identify patients at high risk for SDs. A specific example cited was the recent work using scalp EEG to identify subarachnoid hemorrhage patients who will develop delayed cerebral ischemia; the EEG changes are likely caused by SDs or at least identify those patients who are likely to develop them. This high-risk group could then be targeted without invasive monitoring in a clinical trial. Such an approach would necessarily include some patients who did not have SD, but on the other hand would be applicable to wider patient populations, particularly those without monitoring. There was broad consensus that work to identify risk factors and surrogate markers of SD should be prioritized for future studies.

## Outcome Measures

A final topic of discussion was the choice of appropriate outcome measures to indicate treatment efficacy. Improvement in a standardized clinical measure such as the Glasgow Outcome Score or modified Rankin Scale was discussed as the ultimate requirement, but several considerations reduced enthusiasm for such generalized measures as near-term goals. One consideration was small patient numbers, as highlighted above. Another was that SDs relate more to specific lesions, and that based on the lesion size and location, they may not relate to gross functional outcomes in many patients. For instance, a right frontal lesion may not significantly affect functional outcome, but a small dominant temporal lesion with expansion into language regions could theoretically result in a preventable aphasia. A third concern was that outcomes would be strongly influenced by physician interventions, practice variability, and other injury factors that typically are not well controlled in clinical trials. Discussants thus urged careful documentation of sedation medications, blood pressure medications, and nimodipine in particular, in both observational and interventional trials, and warned about implications of such variability for translation of pre-clinical results. Clinical variables should be controlled and documented as much as possible, deviations from normal should be corrected, and normalization of data and results to a patient’s baseline may help. Conversely, pre-clinical studies should aim to replicate clinical conditions, for instance, in choice of anesthetics.

As an alternative to global outcome measures, it was proposed that imaging measures of intracranial lesions be used to assess benefit of SD-targeted treatments. The rationale for this approach is that the outcome measure should be linked closely to the targeted mechanism, and SDs are associated with lesion development and edema. Ideally, early and late imaging should be performed so that a patient can serve as his/her own baseline, and changes can be assessed over the period of treatment and monitoring. It was cautioned that this entails a large work burden to obtain images and quantify lesions, particularly when lesions are smaller and require magnetic resonance imaging. However, this may be the highest quality science and could be implemented in a smaller feasibility study.

## Other Considerations

Support for an interventional trial was not unanimous, as one participant questioned the ethics of withholding treatment in some patients, as would be necessary in a control arm. It was suggested that watching a patient with multiple SDs that induce a progressive flattening of the ECoG recordings would be similar to watching a patient with intracranial pressure rising to 40 or 50 mmHg without taking appropriate countermeasures. A few other concerns for trial design were also expressed. One was based on a case presented at the meeting, in which SDs were effectively suppressed at the location of a subdural ECoG strip, yet continued to occur at a separate location monitored with a depth electrode. This raised the possibility of false positives in SD suppression, and suggested the need for more widespread ECoG monitoring of affected tissue than is provided by a single subdural strip. Another participant, in a related comment, suggested that the location of electrode strip placement should be standardized, as practice variance between centers could impact results.

## Conclusions

There was fear that accumulating clinical evidence for the adverse effect of SDs, at least some patterns in some patients, together with increasing clinical use of ketamine, could lead to an overly enthusiastic and oversimplified view that “all SDs are bad and should be treated with ketamine.” Without an adequate evidence base, this scenario could be bad for patients and damaging to this promising field of translational neuroscience. There was consensus for the need for the field to move forward and scientifically determine the best method and clinical benefit of interventions targeted at SD pathology. The clinical science of SD represents one of the most promising opportunities for implementing a precision medicine approach to neuroprotection in acute brain injury, yet faces some acknowledged pitfalls, including challenges in generalizability. There was considerable support for studies utilizing real-time SD monitoring, and given the complexities of such a trial conducted at multiple centers, there was agreement that a feasibility study should be considered first. A specific trial discussed was the use of ketamine, in combination with midazolam, as the preferred sedative for patients in whom SDs are observed. Nonetheless, a diversity of opinions were expressed in this discussion, reflecting the wide range of expertise and the early-stage development of the field.

